# Influence of Healthcare Delivery Type on Patients’ Mental Health: Is Hospitalization Always a Stressful Factor? Can Allostatic Load Help Assess a Patient’s Psychological Disorders?

**DOI:** 10.3390/clinpract14030079

**Published:** 2024-05-30

**Authors:** Ana María Gómez García, Eduardo García-Rico

**Affiliations:** 1 Internal Medicine Unit, Hospital Universitario HM Madrid, 28015 Madrid, Spain; 2 Facultad HM de Ciencias de la Salud de la Universidad Camilo José Cela, 28010 Madrid, Spain; 3 Medical Oncology Unit, Hospital Universitario HM Torrelodones, 28250 Madrid, Spain

**Keywords:** COVID-19, anxiety, hospitalized, outpatient, allostatic load

## Abstract

Background: Psychological distress is a predictor of future health and disease data, with consequent implications for both the patient and the healthcare system. Taking advantage of the unprecedented situation caused by the COVID-19 pandemic we aimed to assess whether the type of medical care received by patients during the initial months of the pandemic influenced their evolution, particularly at the psychological level. Additionally, we investigated whether allostatic load was associated not only with physical but also psychological alterations. Methods: All the patients diagnosed with COVID-19 infection at HM Madrid Hospital during the month of March 2020 were studied, both those hospitalized (110) and those treated on an outpatient basis (46). They were psychologically evaluated using the Profile of Mood States (POMS) test. We calculated the allostatic load using different laboratory parameters. Results: Outpatient patients had significantly higher scores than hospitalized ones in Tension-Anxiety (52 ± 19.3 vs. 38 ± 4.3; *p* < 0.001). So, 36.9% of the outpatient patients exhibited anxiety. Allostatic load has not been correlated with patients’ psychological alterations. Conclusions: Psychological distress of outpatient patients should be taken into account in their management to improve mental health planning. This knowledge could provide comprehensive care to patients including their mental health, in the face of subsequent epidemics/pandemics.

## 1. Introduction

In December 2019, Wuhan (China) reported the first cases of contagion with a new coronavirus, called SARS-CoV 2 (COVID-19). The virus rapidly spread throughout Southeast Asia and reached Central Europe [1]. On 1 February 2020, the World Health Organization (WHO) declared it a pandemic, since at that time it affected more than one hundred countries, with more than 100,000 cases declared [2]. Due to the situation, the Spanish Government declared a state of alarm and confined the entire population to their homes on 14 March 2020. The COVID-19 virus created an unprecedented situation regarding the efforts to control spreading and to avoid its high mortality rate.

According to figures from the Spanish Department of National Security, the number of confirmed cases on 18 April 2020 was 200,210, with the number of deaths reaching 20,852. Spain was the European country with the highest number of reported cases and the second country with the highest number of deaths after Italy.

During the early months of the pandemic physical manifestations of the infection, at different levels [3], were described in the science community, but those related to mental disorders (in patients infected by COVID-19 and in the general population, in relation to quarantine) were difficult to find. Most of these studies were focused only on healthcare personnel (doctors, nurses, etc.), pregnant women, patients with pre-existing mental illnesses, students, but only a few of them were carried on COVID-19 patients. Therefore we knew little about the psychological effects on patients. According to Dorman-Ilan et al. [4] the emotional impact of the pandemic itself was added. It was not uncommon for confirmed or suspected COVID-19 hospitalized patients to suffer great psychological pressure and other mental health problems (feelings of loneliness, denial, anxious symptoms, sadness, insomnia, hypersomnia, despair, mood changes, irritability, etc.) [5,6,7,8,9].

Generalized outbreaks of infectious diseases are not only associated with physical symptoms but also with psychological distress and symptoms of mental illness. Similar to natural disasters, these situations are associated with high levels of anxiety and distress, that represent some of the precursors of post-traumatic stress disorder (PTSD). In fact, previous studies conducted in past epidemics and pandemics (SARS, Ebola, etc.) have shown that the epidemic spread of viral diseases can be related to anxiety, depression, distress, sleep disorders, PTSD, not only due to their medical impact but also due to the impact of quarantine measures aimed at reducing the outbreak [10,11].

According to data from a study conducted from March to May 2020, 46% of the Spanish population reported an increase in psychological distress during the lockdown, and 44% indicated that their optimism and confidence had decreased [12].

According to the Center for Sociological Research (CIS), from the beginning of the pandemic until the present, 6.4% of the population had sought help from a mental health professional for some type of symptom, with the highest percentage being 43.7% for anxiety and 35.5% for depression. According to this same study, 5.8% of the population had received psychopharmacological treatment, with anxiolytics (58.7%) and antidepressants (41.3%) being the most common. 68.7% had taken them for more than 3 months.

From the experience of these epidemics, it has been described that infected patients experience different mental health problems like anxiety, depression, post-traumatic stress disorder, sleep disorders to varying degrees, even after being discharged [13]. Even those having fully recovered physically, even a year later they still had deteriorating mental health [14].

More than six months after being discharged from the hospital, one-third of patients who survived severe acute respiratory syndrome (SARS) and Middle East respiratory syndrome (MERS) coronavirus infections experienced persistent symptoms of anxiety (30%) or depression (33%), and 39% were diagnosed with post-traumatic stress disorder [15].

This has also happened in the COVID-19 pandemic, where a significant number of patients who had already recovered from the disease suffered stress for weeks, as well as depression, anxiety and fear. Many of the COVID-19 survivors had to face not only physical but also emotional and psychological consequences. For example, months after the onset of symptoms, many patients still had some symptoms such as asthenia, muscle weakness, sleep disorders, anxiety and depression [16], which are the most common.

Public health policies, especially at the beginning of the pandemic, focused on preventing the spread of the disease and treating cases, with attention centered on pharmacological measures and neglecting the impact on mental health. Months later, we became aware of the psychological sequelae in patients who suffered from the disease and in the general population.

A systematic review and meta-analysis of psychiatric manifestations associated with SARS/MERS and SARS-CoV-2 concludes that post-traumatic stress disorder is very prevalent in the acute phase (32%), followed by memory deficits (19%), irritability (12.8%), anxiety (12.3%), insomnia (12%), and depression (19.5%). In the convalescent phase, the meta-analysis showed that the prevalence of post-traumatic stress disorder was 32.2%; depression was 14.9%; and anxiety disorders were 14.8% [17].

These data are of great importance since psychological distress is a predictor of future health and disease data. In some patients, who initially had an adaptive disorder, this will eventually become a serious mental disorder that will persist over time, with consequent implications for both the patient and the healthcare system. Thus it must be taken into account to improve mental health planning and implement preventive measures in subsequent epidemics/pandemics [18], in the management of infected patients, among other things.

There are very few studies comparing the psychological impact on patients infected with SARS-CoV-2 who are hospitalized and those managed on an outpatient basis at home, and none comparing anxiety in both groups. It seems logical to assume that this impact would be greater in hospitalized patients, as it is assumed that they are hospitalized based on criteria such of age, severity (clinical, radiological, etc.), and also the simple fact of hospitalization has been shown to be associated with mental disorders by itself (anxiety, depression, delirium, psychomotor agitation syndrome, etc.). Any healthcare professional who cares for patients admitted to a hospital is aware of the potential deleterious effect that a hospital admission has on them, both physically and mentally. Anxiety and depression are common in hospitalized patients, described in previous studies that confirmed that they are independent of the severity of the illness that led to admission [19]. Gullich et al. estimate the prevalence of psychiatric disorders in general hospitals between 30–60%, being the most frequent, anxiety and depression [20].

However, we believe that in this extraordinary situation, patients managed on an outpatient basis received an equal or greater psychological impact than those hospitalized, given the uncertainty surrounding the disease especially during the initial months, with alarming news regarding its mortality, the collapse of the healthcare system, and the shortage of resources.

Therefore, looking ahead to the management of similar situations in a future, having data on the mental health of infected patients and the possible impact on it of the type of medical care received it is of great interest in order to provide the necessary knowledge to develop strategies (preventive and/or therapeutic) that can reduce it, and thus provide comprehensive care to patients including their mental health, in the face of future subsequent epidemics/pandemics.

The objective of the study was to analyze and describe the emotional impact and potential psychopathological consequences of COVID-19 infection, and to compare this impact between hospitalized patients and patients managed on an outpatient basis. This way, it could be possible to identify the vulnerable groups in need of greater psychological health support, in similar situations. The hypothesis was considered that outpatient patients exhibited a higher prevalence of psychiatric pathology compared to those hospitalized. The relationship between the number of emergency room visits by outpatient patients and Tension-Anxiety measured in the POMS test was also analyzed.

We have not found previous mental health studies in epidemics that compare anxiety levels between these two patient groups. In a complex health situation, there is a tendency to prioritize hospitalized patients, assuming their greater severity, when patients treated at home may experience an even greater emotional impact. This study suggests that, in a similar situation, special attention should be paid to the mental health of these patients, and the possibility of developing psychiatric pathology should be considered early not only in hospitalized patients but also in outpatient patients, as early diagnosis and treatment could prevent other adverse outcomes for mental health [21,22,23]. In view of similar situations in the future, it would be advisable to develop tools that allow for the simple assessment of patients’ psychological status (for example, online self-reports) to identify the presence and severity of symptoms, their needs, and to provide appropriate treatment to minimize the impact on their health [6].

Another objective of the study was to analyze whether allostatic load was related to the psychological alterations observed in patients and assessed using the POMS test. Using the concept of allostatic load we also evaluated different clinical and laboratory parameters, and using them we calculate its value, with the aim of assessing its association with different psychological factors evaluated through the POMS test. Allostasis is the process of adaptation to a stressful situation Allostasis is the process of adaptation to a stressful situation, and its mediation, and its mediators are the hypothalamic-pituitary-adrenal axis and the autonomic nervous system. Allostatic load reflects the wear and tear or exhaustion of allostatic systems, and in the long term is a cause of both organic and psychic pathology. The concept of allostasis has not been previously associated with COVID-19 but has been linked to other diseases [24], with elevated allostatic load being associated with a higher risk of all-cause mortality [25] and multiple negative health outcomes, both physical and mental. The increase in allostatic load, which can occur through the accumulation of stress over the lifespan, can significantly alter the functioning of different physiological systems. There is evidence that allostatic overload leads to hypercortisolemia, dysregulation of the hypothalamic-pituitary-adrenal (HPA) axis, elevated proinflammatory cytokines and chemokines, reduced synaptic plasticity, persistently activated microglia, and dysbiotic intestinal microbiota [26].

A complete theoretical framework for allostatic load has not yet been developed, although it has been established as a diagnostic tool to assess toxic stress in various contexts. The literature on this topic has increased over the past thirty years, highlighting the utility of this easily calculable marker (although consensus is still lacking), which has shown greater utility than various individual biomarkers in predicting disease severity and patient outcomes.

## 2. Materials and Methods

### 2.1. Study Design and Participants

This observational retrospective study was conducted on patients hospitalized at Hospital Madrid diagnosed with COVID-19 infection from 1 March 2020 to 31 March 2020, and on patients diagnosed during the same month in the Emergency Department and managed on an outpatient basis. Patients diagnosed with COVID-19 infection by polymerase chain reaction (PCR) in nasal and pharyngeal swabs, and/or clinical and/or compatible pulmonary radiology, who agreed to participate voluntarily and were over 18 years of age, were included. On the other hand, those patients with previously diagnosed severe psychiatric illness (e.g., schizophrenia, bipolar disorder, anxiety disorder, depressive disorder), any type of cognitive impairment, inability to complete questionnaires, and those on treatment with drugs that could cause side effects associated with depression, anxiety, insomnia (e.g., steroids) were excluded.

The decision to hospitalize a patient or treat them on an outpatient basis (at home) was made by the emergency room physicians based on severity (clinical, radiological) and bed availability, without standardized criteria at that time.

We recruited 46 patients treated in the Emergency Department and managed on an outpatient basis, and 110 hospitalized patients (the entirety of patients treated in the hospital during the described period). According to our pre-registered criteria, 7 patients were excluded due to pre-existing mental pathology (2 anxiety, 3 depression, 2 cognitive impairment).

As this is an observational study and not randomized, outpatient and hospitalized patients may differ in some characteristics. However, for the majority of variables, there were no differences between the two groups. A multivariate analysis using multiple linear regression was performed to compute β-coefficients (adjusted for age) and their corresponding 95% confidence intervals for outpatient and hospitalized patients across each mental health measure. The findings are presented in Table 1. Notably, the dimensions exhibiting significant differences between the two groups remain consistent with those previously reported (Tension-Anxiety, Anger-Hostility, and Fatigue).

### 2.2. Materials and Measurements

The data collection was performed through the completion of two questionnaires by the patients, one for psychological assessment and another for evaluating demographic variables. These questionnaires were completed by the patients months after the infection, during a follow-up appointment at Hospital Madrid. The study took approximately 20 min to complete; 10 min for the demographic test and 10 min for the POMS test.

The demographic test consisted of 26 items. Demographic parameters such as age, gender, socioeconomic data including housing conditions, educational level, family income, employment status before and during the pandemic lockdown, changes in habits, psychological problems, etc., were evaluated.

Psychological assessment was carried out in both groups (outpatient and hospitalized patients) using the POMS test, which measures various aspects of people’s mood. It is one of the most widely used instruments in psychology for measuring feelings, affects, and moods. The POMS is a very popular tool among sports psychologists in particular. It consists of 58 items and evaluates the factors of Tension-Anxiety, Depression-Dejection, Anger-Hostility, Vigor-Activity, Fatigue-Inertia, and Confusion-Disorientation. It is a versatile test that has been the subject of different versions aimed at simplifying its content, either by measuring all factors with fewer items or by using fewer factors. Originally developed for non-psychiatric populations and later for psychiatric populations, its field of application has expanded to include different populations, including the elderly. McNair et al. created the POMS in 1971 as a measure of psychological progress in patients undergoing treatment with medication and/or psychotherapy. The standard instruction protocol employs the expression ‘how have you felt during the past week, including today? The test creators chose to refer to the past week as they considered it a time period long enough to capture typical and enduring emotional reactions of individuals to daily life events, yet short enough to assess acute effects of a treatment. However, they also indicated the possibility of using alternative instructions depending on the study’s purpose. Subsequent research has examined the consequences of changing the reference times in mood assessment. According to Watson, the structure of affective factors, positive and negative, emerged independently of the specified time frame, and Winkielman, Knauper, and Schwarz assert that individuals can adjust their reference periods to rate their moods. A recent study by Andrade et al. evaluated the invariance of the POMS questionnaire across response times and type of instruction, as well as administration circumstances, which are relevant aspects for a meaningful interpretation of self-report scores. It appears that the use of one type or another of instruction does not compromise the overall psychometric quality of the measure. For this reason, this questionnaire was chosen to assess the mood of patients, as the study was conducted months after experiencing the infection, and an advantage of this test is that it allows for the measurement of both current and past moods, thus not excluding the possibility of a bias related to the recall of the stressful event.

In this study, we used the translation of the POMS carried out by the Department of Sports Psychology at the High Performance Center of San Cugat del Vallés. This instrument has been validated by the Sports Psychology Research Unit. The translation consists of 58 adjectives that make up the 6 factors obtained by the scale authors. The reliability coefficients (Cronbach’s Alpha) of the scale were satisfactory. Their range extends between 0.80 and 0.90.

Each one was assessed using the Likert scale, and scores for each factor were obtained by summing the scores of the items that compose it. The global score was calculated by summing the scores in the factors, subtracting the corresponding Vigor score. These values were referenced to normality criteria specified below (determined by the authors of the test):

Cut-off points:
TENSION/ANXIETY. Normal 39–59
DEPRESSION. Normal <45
ANGER. Normal <53
VIGOR. Normal >57
FATIGUE. Normal <50
CONFUSION. Normal <50

Means and standard deviations were also calculated for each factor in the group, as well as the number and percentage of patients who exhibited abnormal values for each factor.

With regard to the calculation of allostatic load, there is no consensus on the best formula. In this study, the various clinical and laboratory parameters were obtained when assessed in the Emergency Department and upon admission to the hospital, and, to simplify the analysis, patients were divided according to the following normality criteria: systolic blood pressure (SBP) > 140, diastolic blood pressure (DBP) < 90, heart rate (HR) > 100, leukocytes > 11,000 or < 4400, lymphocytes > 3400 or < 1200, D-dimer > 500, lactate dehydrogenase (LDH) > 480, C reactive protein (CRP) > 5, ferritin > 150, procalcitonin > 0.5, fibrinogen > 450, lactate > 2, interleukin-6 (IL-6) > 1.8. We chose these parameters because they were easy to obtain and were available for all patients. There is still no standardized method for calculating the allostatic load nor specific validated tools to measure it. It is important to note that the study does not consider other clinical parameters such as respiratory stress or vascular disorders that are indicative of the disease. In this case, a value of 1 was assigned to each altered parameter and 0 to each normal parameter, and the sum of the scores obtained for each parameter was performed for each patient.

### 2.3. Statistical Analysis

A descriptive analysis was conducted, presenting categorical variables as frequencies and continuous variables as mean and standard deviation or median and interquartile range, depending on their distribution. Normality of continuous variables was assessed using the Kolmogorov-Smirnov test or Shapiro-Wilk test.

Categorical variables were compared between hospitalised and outpatient groups using the chi-square test or Fisher’s exact test. Similarly, quantitative variables were compared using Student’s t-test or Mann-Whitney U-test, based on their distributions. Statistical analyses were performed using SPSS 29 (IBM Corp., Armonk, NY, USA) or GraphPad Prism version 8.0.2 for Windows (GraphPad Software, San Diego, CA, USA). A *p*-value < 0.05 was considered statistically significant.

## 3. Results

The majority of the evaluated patients were elderly adults. The outpatient group showed a wider age range, ranging from 25 to 82 years, compared to the hospitalized group where half of the patients were aged over 54 years and 25% of them were even older than 65 years. The outpatient population appeared to be equivalent in terms of gender, with a slightly higher proportion of women, whereas in the hospitalized patient group the results indicated a higher proportion of women (64.5%) than men (35.5%). The vast majority of evaluated patients had a high level of education. This is reflected in the income and employment status of the group, with a socioeconomic status ranging from medium to high. The results indicate that the pandemic had a heterogeneous effect on the patients’ employment status. Statistically significant differences were found in age. It can be observed that outpatient patients had a significantly younger age compared to hospitalized ones (52.5 ± 15.1 vs. 60.5 ± 16.2; *p* = 0.004) (Table 2).

**Figure 1:**
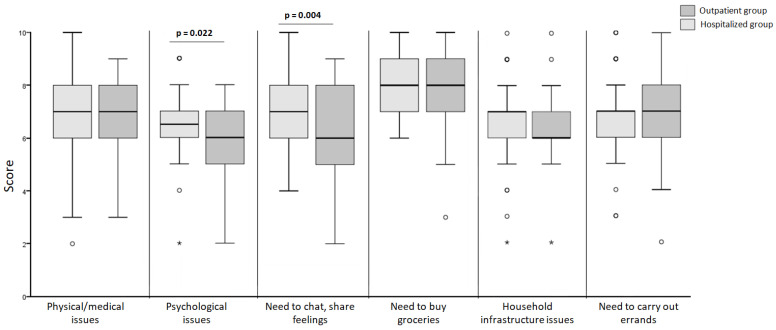
Comparison of perceived support in different contexts (the points mark the outlier values. The asterisk mark the extreme values).

In relation to the availability of assistance for various daily activities as well as adequate information about the disease, there were statistically significant differences in the perceived support patients believed they could rely on regarding psychological issues and for chatting and sharing emotions. It can be observed that outpatient patients had significantly lower scores in these aspects compared to hospitalized ones (6 ± 2 vs. 6.5 ± 1; *p* = 0.022 y 6 ± 3 vs. 7 ± 2; *p* = 0.004; respectively) (Figure 1).

It appears that there was a trend for outpatient patients to require more psychological support during the lockdown than hospitalized patients, although not reaching statistical significance (21.7% vs. 9.1%, *p* = 0.059) (Table 3).

**Figure 2:**
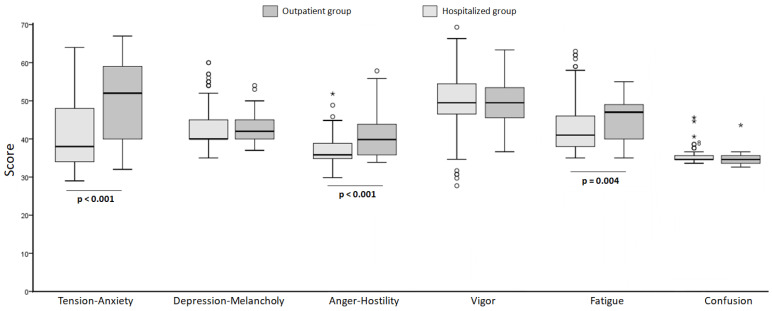
Comparative analysis of parameters related to the POMS test in hospitalized and outpatient patients (the points mark the outlier values. The asterisk mark the extreme values).

A comparative analysis of parameters related to the POMS test was conducted between patients treated on an outpatient basis and those hospitalized (Figure 2). The overall Cronbach’s alpha value for the Profile of Mood States (POMS) was 0.752, indicating good reliability.

We calculated the median score, the 25th percentile and the 75th percentile for each parameter of the test. The results indicated that in the outpatient group 36.9% of the patients exhibited anxiety or tension at the time of the test. In the hospitalized group, the results indicated a tendency toward low values in the Tension-Anxiety rating, with only 5 patients (4.5%) scored above the upper limit of the normal range (59 points).

For Depression-Melancholy, it was observed that among outpatient patients the results indicated that 15.2% of the evaluated patients exhibited depression or melancholy. In the hospitalized patients the results indicated that a significant proportion of hospitalized patients suffered from depression or melancholy. Depression levels were noticeably higher in outpatient patients compared to hospitalized ones. However, when comparing the values using the Mann Whitney test, it was shown that the difference between the medians was not statistically significant. The value of the test (Mann Whitney U) was 2209, with a *p*-value of 0.235 (greater than 0.05).

Similarly, for Anger, the values in outpatient patients indicated that the majority of the studied population had normal values for this parameter. Only 3 patients had values above 53 (the cut-off point), corresponding to 6.5%. The majority of hospitalized patients did not experience anger.

Regarding Vigor, in outpatient patients 93.4% had values below the norm (57), indicating that this parameter was altered. In hospitalized patients, only 20 of them (corresponding to 18.18% of the evaluated group) had values ≥ 57. A trend towards lower vigor in outpatient patients is observed, although there were no significant differences between the groups. The data described indicate that there were no differences between the median values for vigor in outpatient compared to hospitalized patients, because the *p*-value was above 0.05.

Regarding Fatigue, in outpatient patients, 15.2% of them had values above 50, indicating reported fatigue. In this case, the values corresponding to the Fatigue indicator in outpatient patients were higher than those observed in hospitalized patients. The difference between the median values of the groups was statistically significant (*p* < 0.05).

When Confusion was studied, no abnormal values were observed in either group.

In summary, it can be observed that outpatient patients had significantly higher scores than hospitalized ones in Tension-Anxiety (52 ± 19.3 vs. 38 ± 4.3; *p* < 0.001), Anger-Hostility (40 ± 8.3 vs. 36 ± 4; *p* < 0.001), and Fatigue (47 ± 9.3 vs. 41 ± 8.3; *p* = 0.004).

The relationship between the number of emergency room visits by outpatient patients and Tension-Anxiety measured in the POMS test was also analyzed. The median values obtained in the POMS test in patients according to the number of emergency room visits were determined. It was observed that the median values for Tension-Anxiety measured in the POMS test were higher in patients who visited the Emergency Room more than 2 times. The results observed in the Table 4 confirm the positive relationship between Tension-Anxiety and the number of Emergency Room visits (39 ± 16.5 vs. 52 ± 10 vs. 60 ± 6; *p* < 0.001). They also show that the same association was observed for Depression-Melancholy (40 ± 2.3 vs. 43 ± 5 vs. 44 ± 5.5; *p* = 0.009) and for the feeling of Anger-Hostility (36.5 ± 6.3 vs. 40 ± 10 vs. 43 ± 5.5; *p* = 0.014), while there was an inverse relationship with Vigor (54 ± 6.3 vs. 49 ± 7 vs. 49 ± 9; *p* = 0.008).

These results indicate that patients who are sick at home, get worse clinically, and they suffer serious mood disturbances (Table 4). In fact, 25 out of 46 patients managed on an outpatient basis stated they believed they would have been better off if they had been hospitalized, representing over 50%. Many of them visited the Emergency Department multiple times (15 on two occasions, 13 on 3–4 occasions).

The different clinical and analytical parameters evaluated are presented below (Table 5), as well as the comparison of allostatic load values in both groups (Table 6).

It can be observed that hospitalized patients had a higher score in allostatic load than outpatient patients (6 ± 2 vs. 3 ± 2; *p* < 0.00). The majority of outpatient patients exhibited an allostatic load in the range of 2–4 points. The maximum possible value with the evaluated parameters was 11 points, so it can be considered that the allostatic load in this group was low. Conversely, among hospitalized patients the most frequently observed allostatic load scores were between 5–8, as expected, being higher among the hospitalized.

When allostatic load was evaluated in relation to the factors analyzed in the POMS test it significantly and inversely correlated with vigor, although statistically significant associations were not observed with any of the other parameters measured in the POMS test. On the other hand, when analyzing the components of allostatic load in relation to vigor, it was observed that abnormal diastolic pressure decreased vigor in these patients.Principio del formulario.

## 4. Discussion

Among outpatient patients, it was found that 15.2% of evaluated patients presented depression or melancholy, compared to 22.9% of hospitalized patients, indicating a trend towards higher depression among hospitalized patients. However, the median values of the scores for depression and melancholy were statistically comparable between the two groups. The literature does not provide comparative studies on depression levels between hospitalized and non-hospitalized patients during the course of the disease. Nevertheless, some follow-up studies of survivors from 1 to 3 months after hospitalizations for COVID-19 have reported significant symptoms of post-traumatic stress, where depression has been one of the triggering factors. It is important to note that non-hospitalized individuals constitute a larger patient group than hospitalized ones [27,28,29].

Regarding anxiety, it was observed that 51.8% of the hospitalized patients had scores below the established cut-off point, while in the outpatient group, it was 36.9%.

In terms of vigor, 93.4% of outpatient patients had values below the normal cutoff point, indicating that patients perceived a lack of energy in their daily lives. Similarly, in the hospitalized group the majority of patients had values outside the norm for this parameter. Only 18.18% of the evaluated group had normal values (≥57) for this parameter. Regardless of the type of care, fatigue was consistently observed in a significant proportion of patients, with 15.2% among outpatient patients and 18.18% among hospitalized patients. However, the median values for fatigue were significantly higher among the outpatient group, indicating that regardless of the number of patients, fatigue was more intense in this group compared to hospitalized patients.

In comparison, outpatient patients exhibited more anxiety and fatigue than hospitalized patients, but did not differ in terms of depression, vigor, or confusion, with the values of these indicators being comparable between the two groups. It was significant that more than 50% of outpatient patients expressed the opinion that they would have been better off if they had been hospitalized.

This study explored various aspects of psychological well-being, including anxiety, depression, fatigue, vigor, anger, and confusion, in relation to the illness using the POMS test, and compared them between hospitalized and non-hospitalized patients. The results revealed significantly higher scores in the outpatient group compared to the hospitalized group, indicating greater anxiety and tension among outpatient patients within the context of COVID-19 care. The literature presents limited studies addressing levels of anxiety, tension, or stress among the non-hospitalized infected population, and the findings have been controversial [30,31,32].

There are studies that have shown hospitalized patients tend to exhibit higher levels of anxiety and a greater risk of post-traumatic stress than patients who have not required hospitalization [33]. These findings have significant implications for health. For instance, depression in hospitalized patients complicates treatment adherence, slows patient recovery, alters disease prognosis and course, increases mortality risk, prolongs hospitalization duration, and consequently escalates the economic burden of the illness. Other studies contribute to reaffirming the importance of anxious and depressive symptomatology, finding that patients frequently experience elevated stress levels while hospitalized. Nevertheless, the results obtained in this study show that hospitalized COVID-19 patients felt their quality of life was better compared to the perceived health well-being of those not hospitalized. This could be explained by social support from family and friends, easy access to medical exams and mental health care, and a potential shift in perspective after recovering from COVID-19, resulting in psychological growth and increased resilience [34]. Thus, COVID patients at home were subjected to a stressful situation that, as the results show, altered their mood, increasing anxiety and depression. It is possible that the uncertainty these patients faced in a pandemic situation worsened their clinical condition and caused greater psychological disturbances (as they did not feel protected by the hospital). This could be reflected in the higher recurrence to the emergency department in patients with higher levels of depression as observed in the study.

The importance of this lies in the fact that increased stress hormones exacerbate inflammatory responses by suppressing the humoral and cellular immune systems, and disrupt the balance of proinflammatory cytokines, altering the immune response to the virus and worsening symptoms and prognosis. In the end, there was an increasing of the instances where patients had to resort to visit the emergency room as a reflection of their deteriorating condition [35,36,37]. As previously mentioned, psychological distress is a predictor of future health and disease data.

When allostatic load was assessed in relation to the factors analyzed in the POMS test, a relationship was only found with vigor, not statistically significant with any of the other factors, suggesting that the mood disturbances observed in these patients were independent of the clinical or physiological alterations attributed to SARS-CoV2 infection. Thus, while previous studies demonstrate that higher levels of allostatic load are associated with poor health outcomes, including psychiatric disorders, little is still known about its relationship with the brain and the mechanism by which stress-related biological dysregulations produce harmful effects. In our study, its association with a worse clinical condition of patients has been highlighted, with higher values found in hospitalized patients, but not with their psychological alterations [38,39]. And yet, despite exhibiting lower physiological disturbance, patients managed on an outpatient basis displayed greater psychological disturbance than hospitalized patients.

The sample size of outpatient patients may be a limitation to the study, as well as the fact that the total patient sample is heterogeneous, having been selected solely based on the criterion of treatment in hospital or at home. The time period in which the study was conducted could also be considered short. Deliberately, we decided to evaluate patients treated during the month of March-20 as we considered them the most representative for the study, since we believe that the emotional impact of these patients at that time is not comparable to those treated in subsequent months. At that time, uncertainty was at its peak due to being a new disease with limited data available. It is not possible to rule out the existence of bias related to the recall of the stressful event when trying to evaluate the mood presented by patients months before the test was conducted. The use of a no validated tool to calculate allostatic load is another limitation of our study, as there is still no standard method to calculate it.

However, although our study is limited, it highlights high levels of anxiety among outpatient patients compared to hospitalized patients.

## 5. Conclusions

The results analyzed in this study, along with those observed in other studies, are not conclusive on their own, but they do indicate high levels of anxiety among these patients, especially in outpatient patients, which could even have effects on clinical parameters such as hypertension and influence the course of the disease. Better structured studies are needed to confirm this finding. Psychological distress is a predictor of future health and disease data, with consequent implications for both the patient and the healthcare system. It should be taken into account in the management of patients to improve mental health planning.

In this study, allostatic load has not been correlated with patients’ psychological alterations.

## Figures and Tables

**Table 1:** Multivariate analysis computing β-coefficients (adjusted for age). * β-coefficients were adjusted for age.

	Outpatients vs. Hospitalized		
	Adjusted β-Coefficients *	95%CI	*p*-Value
Tension-Anxiety	8.16	(5.03 to 11.29)	**<0.001**
Depression-Melancholy	−0.340	(−2.17 to 1.49)	0.714
Anger-Hostility	3.69	(2.9 to 5.18)	**<0.001**
Vigor	−0.915	(−3.46 to 1.63)	0.479
Fatigue	2.35	(0.06 to 4.64)	**0.044**
Confusion	−0.506	(−1.17 to 0.153)	0.132

**Table 2:** Comparison of sociodemographic variables in the hospitalized and outpatient patient groups.

		Overall (*n* = 156)	Hospitalized Group (*n* = 110)	Outpatient Group (*n* = 46)	*p*
Median age ± SD (range)		56.1 ± 16.2 (20–98)	60.5 ± 16.2 (20–98)	52.5 ± 15.1 (25–82)	**0.004**
Gender	Male	61 (39.1%)	39 (35.5%)	22 (47.8%)	0.149
	Female	95 (60.9%)	71 (64.5%)	24 (52.2%)	
Civil status	Single	20 (12.8%)	11 (10%)	9 (19.6%)	0.092
	Married	90 (57.7%)	61 (55.5%)	29 (63%)	
	Widower	21 (13.5%)	19 (17.3%)	2 (4.3%)	
	Separated	12 (7.7%)	8 (7.3%)	4 (8.7%)	
	Divorced	13 (8.3%)	11 (10%)	2 (4.3%)	
Educational level	Primary studies.	1 (0.6%)	1 (0.9%)	0	0.444
	Elementary Baccalaureate, School Graduate, primary education completed or equivalent	11 (7.1%)	6 (5.5%)	5 (10.9%)	
	First or second degree vocational training.	19 (12.2%)	16 (14.9%)	3 (6.5%)	
	Bachelor’s degree, Secondary Education studies.	26 (16.7%)	20 (18.2%)	6 (13%)	
	Diploma from university schools, technical architect, technical engineer.	27 (17.3%)	17 (15.5%)	10 (21.7%)	
	University master’s degree/other higher university studies	72 (46.2%)	50 (45.5%)	22 (47.8%)	
Employment status	Salaried public sector employee	13 (8.4%)	9 (8.3%)	4 (8.7%)	0.495
	Salaried private sector	76 (49.4%)	51 (47.2%)	25 (54.3%)	
	Entrepreneur	4 (2.6%)	2 (1.9%)	2 (4.3%)	
	Unemployed	6 (3.9%)	3 (2.8%)	3 (6.5%)	
	Retired	42 (27.3%)	34 (31.5%)	8 (17.4%)	
	Student	2 (1.3%)	2 (1.9%)	0	
	Dedicated to housework.	10 (6.5%)	6 (5.6%)	4 (8.7%)	
	With permanent work Disability	1 (0.6%)	1 (0.9%)	0	
Monthly income	Less than 1000 euros	4 (2.6%)	3 (2.7%)	1 (2.2%)	0.995
	1000–1999 euros	44 (28.2%)	32 (29.1%)	12 (26.1%)	
	2000–2999 euros	64 (41%)	45 (40.9%)	19 (41.3%)	
	3000–3999 euros	30 (19.2%)	20 (18.2%)	10 (21.7%)	
	4000–4999 euros	11 (7.1%)	8 (7.3%)	3 (6.5%)	
	More than 5000	3 (1.9%)	2 (1.8%)	1 (2.2%)	
Changes in the employment situation	Unemployed	63 (40.4%)	48 (43.6%)	15 (32.6%)	0.241
	I lost my job temporarily	1 (0.6%)	0	1 (2.2%)	
	I lost my job permanently	41 (26.3%)	29 (26.4%)	12 (26.1%)	
	I kept the job	51 (32.7%)	33 (30%)	18 (39.1%)	
Kept their job	Worse conditions than before	2 (4%)	2 (6.1%)	0	0.431
	Same conditions	48 (96%)	31 (93.9%)	17 (100%)	
Children		112 (71.8%)	82 (74.5%)	30 (65.2%)	0.238
Nº cohabitants	0	32 (20.5%)	25 (22.7%)	5 (10.9%)	0.592
	1	77 (49.4%)	52 (47.3%)	25 (54.3%)	
	2	27 (17.3%)	19 (17.3%)	8 (17.4%)	
	3	15 (9.6%)	11 (10%)	4 (8.7%)	
	4	4 (2.6%)	3 (2.7%)	1 (2.2%)	
	5	1 (2.2%)	0	1 (2.2%)	
	No response	2	0	2 (4.3%)	

**Table 3:** Psychological support during confinement.

		Overall (*n* = 156)	Hospitalized Group (*n* = 110)	Outpatient Group (*n* = 46)	*p*
Psychological help during confinement	No	133 (85.3%)	97 (88.2%)	36 (78.3%)	0.059
	Yes	20 (12.8%)	10 (9.1%)	10 (21.7%)	
	I prefer not to answer	3 (1.9%)	3 (2.7%)	0	

**Table 4:** Relationship between the number of emergency room visits by outpatient patients and Tension-Anxiety measured in the POMS test.

	1 Visit (*n* = 18)	2 Visits (*n* = 15)	3–4 Visits (*n* = 13)	*p*
Tension-Anxiety (median ± IQR)	39 ± 16.5	52 ± 10	60 ± 6	**<0.001**
Depression-Melancholy (median ± IQR)	40 ± 2.3	43 ± 5	44 ± 5.5	**0.009**
Anger-Hostility (median ± IQR)	36.5 ± 6.3	40 ± 10	43 ± 10	**0.014**
Vigor (median ± IQR)	54 ± 6.3	49 ± 7	49 ± 9	**0.008**
Fatigue (median ± IQR)	46 ± 10	48 ± 9	48 ± 8.5	0.419
Confusion (median ± IQR)	33 ± 2.3	33 ± 2	32 ± 2	0.624

**Table 5:** Comparative clinical and analytical parameters in hospitalized and outpatient patients.

		Overall (*n* =156)	Hospitalized Group (*n* = 110)	Outpatient Group (*n* = 46)	*p*
Systolic Blood Pressure (median ± IQR)		127 ± 31.3	128.5 ± 33.3	125.5 ± 22.3	0.257
	Normal (140 or less)	117 (75%)	79 (71.8%)	38 (82.6%)	0.156
	Elevated (more than 140)	39 (25%)	31 (28.2%)	8 (17.4%)	
Diastolic Blood Pressure (median ± IQR)		73.5 ± 18	76 ± 19.3	69 ± 14.5	**0.008**
	Normal (less than 90)	131 (84%)	89 (80.9%)	42 (91.3%)	0.107
	Elevated (90 or more)	25 (16%)	21 (19.1%)	4 (8.7%)	
Heart Rate (median ± IQR)		84.5 ± 19	86 ± 17.3	79 ± 19.8	**0.003**
	Normal (less than 100)	133 (85.3%)	91 (82.7%)	42 (91.3%)	0.168
	Elevated (100 oo more)	23 (14.7%)	19 (17.3%)	4 (8.7%)	
Leukocytes (median ± IQR)		5435 ± 2493	5385 ± 2632	5618 ± 2118	0.607
	Normal (4400–11,000)	119 (76.3%)	81 (73.6%)	38 (82.6%)	0.230
	Altered (>11,000 or < 4400)	37 (23.7%)	29 (26.4%)	8 (17.4%)	
Lymphocites (median ± IQR)		1049 ± 469	1010 ± 400	1100 ± 478	**0.020**
	Normal (1200–3400)	49 (31.4%)	28 (25.5%)	21 (45.7%)	**0.013**
	Altered (>3400 or <1200)	107 (68.6%)	82 (74.5%)	25 (54.3%)	
D dimer (median ± IQR)		596 ± 622	810 ± 907	429.5 ± 216	**<0.001**
	Normal (<500)	63 (40.6%)	30 (27.5%)	33 (71.7%)	**<0.001**
	Elevated (>500)	92 (59.4%)	79 (72.5%)	13 (28.3%)	
C–reactive protein (median ± IQR)		38.9 ± 68.7	54 ± 80.9	19.6 ± 30.3	**<0.001**
	Normal (<5)	12 (7.7%)	5 (4.5%)	7 (15.2%)	**0.042**
	Elevated (>5)	144 (92.3%)	105 (95.5%)	39 (84.8%)	
Fibrinogen (median ± IQR)		468 ± 301	556.5 ± 290	287 ± 147	**<0.001**
	Normal (<450)	76 (48.7%)	35 (31.8%)	41 (89.1%)	**<0.001**
	Elevated (>450)	80 (51.3%)	75 (68.2%)	5 (10.9%)	
Ferritin (median ± IQR)		532 ± 280	645 ± 968	385.5 ± 244	**<0.001**
	Normal (<150)	1 (0.6%)	1 (0.9%)	0	>0.999
	Elevated (>150)	154 (99.4%)	108 (99.1%)	46 (100%)	
Lactate dehydrogenase (median ± IQR)		402.2 ± 246	484 ± 290	288 ± 124	**<0.001**
	Normal (<480)	96 (61.9%)	51 (46.8%)	45 (97.8%)	**<0.001**
	Elevated (>480)	59 (38.1%)	58 (53.2%)	1 (2.2%)	
Lactate (median ± IQR)		1.2 ± 0.5	1.3 ± 0.7	1.1 ± 0.4	0.058
	Normal (<2)	143 (92.9%)	97 (89.8%)	46 (100%)	**0.034**
	Elevated (>2)	11 (7.1%)	11(10.2%)	0	

**Table 6:** Comparative allostatic load in hospitalized and outpatient patients.

		Overall (*n* = 156)	Hospitalized Group (*n* = 110)	Outpatient Group (*n* = 46)	*p*
Score Allostatic Load (median ± IQR)		5 ± 2	6 ± 2	3 ± 2	**<0.001**
	1	3 (1.9%)	1 (0.9%)	2 (4.3%)	**<0.001**
	2	18 (11.5%)	6 (5.5%)	12 (26.1%)	
	3	17 (10.9%)	6 (5.5%)	11 (23.9%)	
	4	28 (17.9%)	13 (11.8%)	15 (32.6%)	
	5	27 (17.3%)	25 (22.7%)	2 (4.3%)	
	6	30 (19.2%)	26 (23.6%)	4 (8.7%)	
	7	16 (10.3%)	16 (14.5%)	0	
	8	13 (8.3%)	13 (11.8%)	0	
	9	2 (1.3%)	2 (1.8%)	0	
	10	2 (1.3%)	2 (1.8%)	0	

## Data Availability

The data that support the findings of this study are available from the corresponding author, Ana María Gómez, upon reasonable request.
